# Risk-taking and self-harm behaviors as markers of adolescent borderline personality disorder

**DOI:** 10.1007/s00787-023-02353-y

**Published:** 2024-01-09

**Authors:** Yasmine Blaha, Marialuisa Cavelti, Stefan Lerch, Annekatrin Steinhoff, Julian Koenig, Michael Kaess

**Affiliations:** 1 University Hospital of Child and Adolescent Psychiatry and Psychotherapy, University of Bern, Bern, Switzerland; 2 Department of Child and Adolescent Psychiatry, Psychosomatics and Psychotherapy, Faculty of Medicine and University Hospital Cologne, University of Cologne, Cologne, Germany; 3 Department of Child and Adolescent Psychiatry, Centre for Psychosocial Medicine, University Hospital Heidelberg, Heidelberg, Germany

**Keywords:** Borderline personality disorder, Adolescence, Risk-taking behaviors, NSSI, Suicide attempt

## Abstract

Adolescence is a critical period for early identification and intervention of borderline personality disorder (BPD). Risk-taking and self-harm behaviors (RSB) have been identified as promising early markers of BPD and correlates of depression in school-based samples. The present study aimed, first, to examine the association between RSB and BPD in a clinical sample of adolescents and, second, to examine whether RSB are also linked to depression. N = 405 participants (82.7% female) were recruited from an outpatient clinic for adolescents with RSB. RSB assessed included truancy, excessive media use, alcohol, tobacco, and illicit drug use, sexual risk-taking, and self-harm behavior. Regression analyses and generalized linear models were performed to examine the associations between individual RSB or patterns of RSB (identified using latent class analysis, LCA) and a diagnosis and severity of BPD or depression. All RSB (except excessive media use) were positively associated with BPD diagnosis and severity. In contrast, only non-suicidal self-injury (NSSI) and suicide attempts were positively associated with depression diagnosis and severity, while illicit drug use was negatively associated with depression severity. The LCA yielded two classes differing in the occurrence of RSB. The high RSB class was more likely to have a BPD diagnosis and greater BPD severity than the low RSB class. Classes did not differ regarding depression diagnosis or severity. As NSSI and suicide attempts were associated with both BPD and depression, the presence of additional RSB, besides self-harm behavior, may represent a specific risk marker for BPD in adolescents.

**Supplementary Information:**

The online version contains supplementary material available at 10.1007/s00787-023-02353-y.

## Introduction

Borderline personality disorder (BPD) is a severe mental disorder characterized by affect dysregulation, interpersonal and identity difficulties, and self-harm behavior [1]. Like most mental health problems, BPD begins in adolescence [2, 3], with symptoms peaking in early adulthood followed by a decline later in life [4, 5]. Prevalence ranges from approximately 3% of adolescents in the general population to ~ 11% of adolescents in outpatient settings and up to 78% of suicidal adolescents in emergency settings [6]. Although the BPD diagnosis in adolescence has long been controversial, there is convincing evidence suggesting that the diagnosis is reliable and valid in adolescence and that its assignment is clinically meaningful, because it paves the way for early intervention [5, 7, 8]. This has also been confirmed by recent guidelines in the field of BPD that summarize the current evidence on early detection and intervention [9]. BPD pathology in adolescence has been found to be associated with lower role and social functioning, life satisfaction, academic and occupational achievement, and partner involvement over the long-term [10]. Increasing evidence indicates that early treatment of BPD can positively impact life trajectories of the affected young people by preventing psychosocial impairment [5, 7, 11]. Therefore, adolescence is a critical time period for early identification of and intervention for BPD to reduce chronification and negative long-term consequences known to result from delayed treatment [12, 13]. Accordingly, identifying risk markers for BPD is an important precondition for early intervention efforts [12].

Risk-taking and self-harm behaviors (RSB) are defined as behaviors that may pose a threat to an individual’s physical or psychosocial development. They include truancy, excessive media use, tobacco, illicit drug and alcohol use, sexual risk behavior, non-suicidal self-injury (NSSI), and suicide attempts [14]. RSB are common in adolescence and may occur for a variety of reasons, including social acceptance within the peer group, as an expression of the search for identity and of the desire for autonomy, or as a coping strategy to deal with the stress of developmental and environmental challenges or even emerging psychological problems [15]. In fact, although RSB in adolescents are normative to some degree, they have been found to be associated with BPD and other neuropsychiatric disorders in adolescent community samples [16, 17]. It was shown in a large population-based adolescent sample that RSB add incremental value to the detection of mental disorders beyond the assessment of psychopathology alone [14]. Previous studies have identified NSSI, risky alcohol use, and sexual risk behavior as promising markers for the development of BPD. Early age at onset and longer duration of NSSI in adolescent patients were found to be predictive of BPD in adulthood [18]. In addition, adolescent NSSI and alcohol consumption among community-dwelling adolescents were shown to be predictive of BPD pathology in the following year [19, 20]. Further, adolescents diagnosed with BPD were more likely to have a greater number of sexual partners [21]. Although a recent study found that inpatients with BPD were not more likely to engage in sexual risk behavior than others, they did have riskier attitudes and norms related to sexual risk behavior [22].

The general problem with identifying risk markers for psychiatric disorders is that they are usually not specific to a particular disorder or clinical entity. Indeed, NSSI and excessive alcohol use are not only associated with BPD but also with depression, which is highly comorbid with BPD [23–25]. In a previous study, for example, adolescents with more depressive symptoms exhibited more risky behaviors such as alcohol or illicit drug use or spent more time in front of a computer than adolescents with fewer depressive symptoms [26].

Taken together, most studies on the association between RSB and BPD have been conducted in non-clinical samples [19, 20, 27]. It is unclear if findings generalize to clinical samples where RSB are more common [28, 29]. Moreover, the question of whether RSB are specific signs of BPD or rather transdiagnostic markers of general psychopathology remains unanswered. Therefore, the aim of the current study was twofold: First, to investigate the associations between individual RSB and BPD diagnosis and severity in a clinical sample of adolescents. Second, to explore whether RSB are exclusively associated with BPD, or also with depression.

## Methods

### Participants and general procedures

Participants were recruited from a specialized outpatient clinic for RSB (AtR!Sk; Ambulanz für Risikoverhalten & Selbstschädigung) at the Department of Child and Adolescent Psychiatry, Center for Psychosocial Medicine, University Hospital Heidelberg, Germany. The service provides low-threshold initial contact, detailed and comprehensive diagnostic assessment of BPD features and evidence-based therapeutic intervention for adolescents with emerging or first presentation BPD [30]. Inclusion criteria for the study were: 12–17 years of age and the presence of at least one of the defined RSB (i.e., truancy, excessive media use, tobacco, alcohol and illicit drug use, sexual risk behavior, NSSI and suicide attempts) at the time of presentation at AtR!Sk. Exclusion criteria were: insufficient German language skills; acute psychotic disorder and/or intention to commit suicide or intention to harm others, requiring immediate inpatient admission; impairment of intellectual functioning; and a diagnosis of bipolar disorder, schizophrenia or schizoaffective disorder. All patients and their legal guardians (if under the age of 16 years) provided written informed consent (or assent, respectively). Assessments were part of the clinical diagnostic assessment at clinic entry. The AtR!Sk cohort study started in 2013 and was completed at the end of 2020. The study was approved by the local ethics committee (ID S-449/2013) and conducted in accordance with the Declaration of Helsinki [31].

### Instruments

#### Demographic data

Demographic data were assessed by a set of standardized questions covering age, sex, and information on school/work, family and living situation.

#### Borderline personality disorder

The German version of the Structured Clinical Interview for DSM-IV-Axis II (SCID-II) [32] was used to assess the BPD criteria according to the DSM-IV that remained unchanged in the DSM-V. BPD diagnosis is met, if at least five of the nine BPD criteria are fulfilled. Although the SCID-II was originally developed for the use in adults, its application in the adolescent population has been validated [32, 33].

#### Depression

To obtain the diagnosis of depression (ICD-10 F32 or F33), the German version of the Mini-International Neuropsychiatric Interview for Children and Adolescents (M.I.N.I-KID 6.0) [34, 35] was used. The M.I.N.I-KID is a brief structured diagnostic interview for children and adolescents aged 6 to 17 that covers the major psychiatric disorders of the DSM-IV and ICD-10. To assess depression severity, the Depressionsinventar für Kinder und Jugendliche (DIKJ) [36] was applied. This 27-items questionnaire assesses symptoms of depression according to the DSM-5.

#### Risk-taking behaviors

Six risk-taking behaviors including truancy, excessive media use, tobacco use, illicit drug use, alcohol use, and sexual risk behavior were assessed, based on evidence from the existing literature (e.g., excessive media use [19, 37], truancy [23], substance use (i.e. tobacco, alcohol, and illicit drug use) [19, 38], sexual risk behavior [27]). Since there are no fixed definitions for when a behavior is considered as risky, the cut-offs were set through consensus between research group members by considering previous publications [14, 19]. The respective items and thresholds for each RSB are described in detail in the Online Resource, Table A.

#### Self-harm behavior

To obtain a detailed assessment of NSSI and suicide attempts, the German version of the Self-Injurious Thoughts and Behavior Interview was applied [39, 40]. It has been validated for the use in adolescents and shows good psychometric properties. In the current study, having at least one day with NSSI or at least one suicide attempt during the past 12 months were used as dichotomous predictor variables in the analyses. A low threshold for NSSI was chosen because recurrent NSSI has not been shown to be a stronger marker than sporadic NSSI [19].

### Statistical analyses

Separate univariate logistic regression analyses were conducted to assess the associations between each individual RSB (risk cut-off met versus not-met) and the presence of a diagnosis of BPD or depression (diagnosis met versus not-met). Odds-ratios (OR) were derived from the respective models as measures of effect size. Generalized linear models (binomial distribution with nine trials) were used to examine the associations between individual RSB (risk cut-off met versus not-met) and the number of fulfilled binominal BPD criteria as a proxy for BPD severity. To examine the relationships between RSB and depression severity, linear regression was applied.

Three sensitivity analyses were conducted. First, as the main analyses were not controlled for age, sex or comorbidity to avoid producing "theoretical" results that would hardly have any application in practice, additional analyses were conducted with age and sex as covariates to gain deeper insight on their influence. Second, to account for comorbidity between BPD and depression, separate bivariate logistic regression analyses were performed to assess the association between each individual RSB and the co-occurrence of BPD and depression. Bivariate logistic regression models the marginal probabilities of BPD and depression—like in the univariate logistic regression—, and additionally estimates the common (joint) odds ratio (COR) for the co-occurrence of BPD and depression, depending on RSB (for details on the statistical approach, see [41] and [42]). We tested if the ratio between the COR for subjects with RSB relative to subjects without RSB is equal to one, to check whether RSB influences comorbidity. Third, to account for the conceptual overlap between BPD criterion 4 (i.e., impulsivity) and alcohol use, drug use, and sexual risk behavior, and between BPD criterion 5 (i.e., self-harm behavior) and NSSI and suicide attempts, respectively, we rerun the univariate regression models with adapted BPD severity scores. For this, when building the sum score, the respective criterion was omitted and the new sum score was multiplied by 9/8 to match the original range of BPD criteria (0–9).

All analyses were adjusted for multiple testing using the Benjamini–Hochberg correction. This correction adjusts the false discovery rate (FDR), leading to more power in finding true positives [43].

To explore patterns of RSB and their potential associations with BPD and depression, a 3-step Latent Class Analysis (LCA) was conducted [44]. First, to estimate the optimal number of classes, models with different numbers of latent classes were specified. Model fit was compared based on fit indices including the Akaike’s Information Criterion (AIC) [45], Schwarz’s Bayesian Information Criterion (BIC) [46], and the relative entropy values [47]. AIC and BIC are both based on the log-likelihood but include an additional penalty for model complexity to prevent overfitting of the data. Smaller values of the AIC and the BIC mean a better model fit. The relative entropy (from here on referred to as entropy) reflects the degree of overlap of the latent classes of a model and can take values between 0 and 1. The higher the value, the better the delineation of the individual classes from each other [48]. Second, each subject was assigned to the most probable class based on modal class assignment. Contrasts of each RSB between classes were performed and adjusted for multiple comparisons using the Šidák correction [49], which controls for the family wise error rate (FWER). Third, the regression models predicting BPD and depression diagnosis and severity were repeated with class as the predictor variable.

All analyses were performed using Stata/SE (17.0, Stata Corp LLC, College Station, TX, USA), except from the bivariate logistic regression that was estimated using the packages VGMA in R version 4.1.2 [50]. An alpha level of 0.05 was applied in analyses.

## Results

### Participants

A total N = 782 patients were invited to take part in the study, of whom n = 678 eventually participated (participation rate of 86.7%). Since the study did not include the assessment of RSB prior to 2015, only data from patients as of that date were included in the current analyses (n = 406 patients). As for one patient only questionnaire data was available, this patient was excluded from the analyses, resulting in a final sample of n = 405. A detailed description of the sociodemographic and clinical characteristics of the total sample (as well as for the subsamples with and without a diagnosis of depression or BPD, respectively) is provided in Table 1.

**Table 1:** Sample characteristics

	Total (N = 405)	No BPD diagnosis (n = 301)	BPD diagnosis (n = 104)	No diagnosis of depression (n = 194)	Diagnosis of depression (n = 211)
Age in years, M (SD)	15.0 (1.5)	14.9 (1.6)	15.4 (1.1)	14.9 (1.6)	15.1 (1.4)
Female sex, n (%)	335 (82.7)	242 (80.4)	93 (89.4)	150 (77.3)	185 (87.7)
BPD, n (%)	104 (25.7)	0 (0.0)	104 (100.0)	44 (22.7)	60 (28.4)
Number of DSM-IV BPD criteria, M (SD)	3.1 (2.1)	2.0 (1.3)	6.0 (1.1)	2.9 (2.3)	3.2 (2.0)
Depression, n (%)	211 (52.1)	151 (50.2)	60 (57.7)	0 (0.0)	211 (100.0)
Severity of depression (N = 349), M (SD)	28.6 (9.6)	27.2 (9.7)	33.2 (7.6)	25.1 (10.2)	31.8 (7.7)
RSB, n (%)					
RSB^a^, M (SD)	3.41 (1.6)	3.15 (1.6)	4.16 (1.5)	3.42 (1.7)	3.40 (1.6)
Truancy	95 (23.5)	60 (19.9)	35 (33.7)	43 (22.2)	52 (24.6)
Media usage	268 (66.2)	196 (65.1)	72 (69.2)	126 (64.9)	142 (67.3)
Alcohol	236 (58.3)	159 (52.8)	77 (74.0)	112 (57.7)	124 (58.8)
Illicit drugs	78 (19.3)	47 (15.6)	31 (29.8)	42 (21.6)	36 (17.1)
Tobacco	203 (50.1)	133 (44.2)	70 (67.3)	105 (54.1)	98 (46.4)
Sexual risk behavior	141 (34.8)	94 (31.2)	47 (45.2)	73 (37.6)	68 (32.2)
NSSI	360 (88.9)	259 (86.0)	101 (97.1)	163 (84.0)	197 (93.4)
Suicide attempts	139 (34.3)	86 (28.6)	53 (51.0)	51 (26.3)	88 (41.7)

*RSB* Risk and self-harm behavior

^a^Mean number of fulfilled RSB, range 0–8

### Associations between RSB and BPD or depression

All RSB, except for excessive media use, were associated with the presence of a BPD diagnosis, increasing the likelihood of being diagnosed with BPD 1.82- to 5.46-fold (see Table 2). Additionally, all RSB were significant predictors of BPD severity, with the presence of RSB increasing the number of BPD criteria by 0.22 to 1.07 criteria. Adolescents who reported at least one occasion of NSSI or one suicide attempt during the past 12 months had a 2.68-fold, respectively, a 2.01-fold increased risk of being diagnosed with depression. Further, depression severity increased by 10.56 points (range of depression severity scale: 0–54) in the presence of NSSI and by 3.86 points in the presence of a suicide attempt. Depression severity decreased by 4.35 points when fulfilling illicit drug use as one of the RSB.

**Table 2:** Separate univariate associations of RSB with BPD and depression diagnosis and severity

	Truancy	Excessive media use	Alcohol use	Illicit drug use	Smoking	Sexual risk behavior	NSSI	Suicide attempt
BPD diagnosis								
OR	2.04	1.21	2.55	2.29	2.60	1.82	5.46	2.60
95% CI	[1.24; 3.34]	[0.75; 1.95]	[1.56; 4.17]	[1.36; 3.87]	[1.63; 4.16]	[1.15; 2.87]	[1.65; 18.01]	[1.64; 4.11]
*p*^*a*^	***0.009***	*0.527*	***0.001***	***0.004***	** < *0.001***	***0.017***	***0.009***	** < *0.001***
Number of BPD criteria								
OR	1.57	1.25	1.73	1.77	1.75	1.58	2.92	1.94
95% CI	[1.34; 1.83]	[1.08; 1.44]	[1.50; 2.00]	[1.50; 2.10]	[1.53; 2.02]	[1.37; 1.82]	[2.22; 3.84]	[1.69; 2.24]
*p*^*a*^	** < *0.001***	***0.007***	** < *0.001***	** < *0.001***	** < *0.001***	** < *0.001***	** < *0.001***	** < *0.001***
Diagnosis of depression								
OR	1.15	1.11	1.04	0.74	0.74	0.79	2.68	2.01
95% CI	[0.72; 1.82]	[0.74; 1.68]	[0.70; 1.55]	[0.45; 1.22]	[0.50; 1.09]	[0.52; 1.19]	[1.38; 5.20]	[1.32; 3.06]
*p*^*a*^	*0.636*	*0.659*	*0.833*	*0.338*	*0.179*	*0.340*	***0.007***	***0.003***
Severity of depression								
* β*	1.25	1.74	0.52	− 4.35	0.38	− 0.94	10.56	3.86
95% CI	[− 1.20; 3.70]	[− 0.37; 3.86]	[− 1.51; 2.55]	[− 7.00; − 1.69]	[− 1.64; 2.40]	[− 3.09; 1.21]	[7.52; 13.60]	[1.76; 5.97]
*p*^*a*^	*0.405*	*0.161*	*0.677*	***0.003***	*0.735*	*0.482*	** < *0.001***	***0.001***

All models without age and sex as covariates

^a^Benjamini-Hochberg corrected *p*-values for multiple testing

When the analyses were repeated with age and sex as covariates (sensitivity analysis 1), the results remained mostly unchanged, with three exceptions (see Online Resource, Table B): Sexual risk behavior was no longer associated with a BPD diagnosis (*p* = 0.149), illicit drug use was no longer associated with depression severity *(p* = 0.100), and NSSI was no longer associated with the diagnosis of depression (*p* = 0.061).

The bivariate logistic regression analysis (sensitivity analysis 2) revealed that none of the RSB were significantly associated with the co-occurrence of BPD and depression, except for suicide attempts (see Online Resource, Table C). The presence of at least one suicide attempt during the past year exhibited the most substantial increase in the probability of a depression alone (probability = 0.424) followed by the probability of the co-occurrence of BPD and depression (probability = 0.209), none of the disorders (probability = 0.194), and of BPD alone (probability = 0.173). For a visual representation of these probabilities, please see Online Resource, Figure A).

When the linear regression models were rerun with adjusted BPD severity scores to account for conceptual overlap between RSB and the BPD criteria 4 and 5 (sensitivity analysis 3), the results did not change (see Online Resource, Table D).

### Patterns of RSB and their associations with BPD and depression

The LCA resulted in a two-class model as the best fitting model (see Table 3). This model had the lowest BIC value and a higher entropy value than the three-class model. The difference in the AIC values between the two-class and the three-class models was very small. Since the BIC penalizes free parameters more strongly than the AIC, slightly more weight was assigned to the BIC.

**Table 3:** Fit indices for LCA models

Nr. of classes	BIC	AIC	Entropy
1	3843.20	3811.17	
2	3604.38	3536.31	0.74
3	3637.81	3533.71	0.60

*BIC* Bayesian information criteria, *AIC* Akaike’s information criteria; Models with one, two and three latent classes are reported

**Fig. 1:**
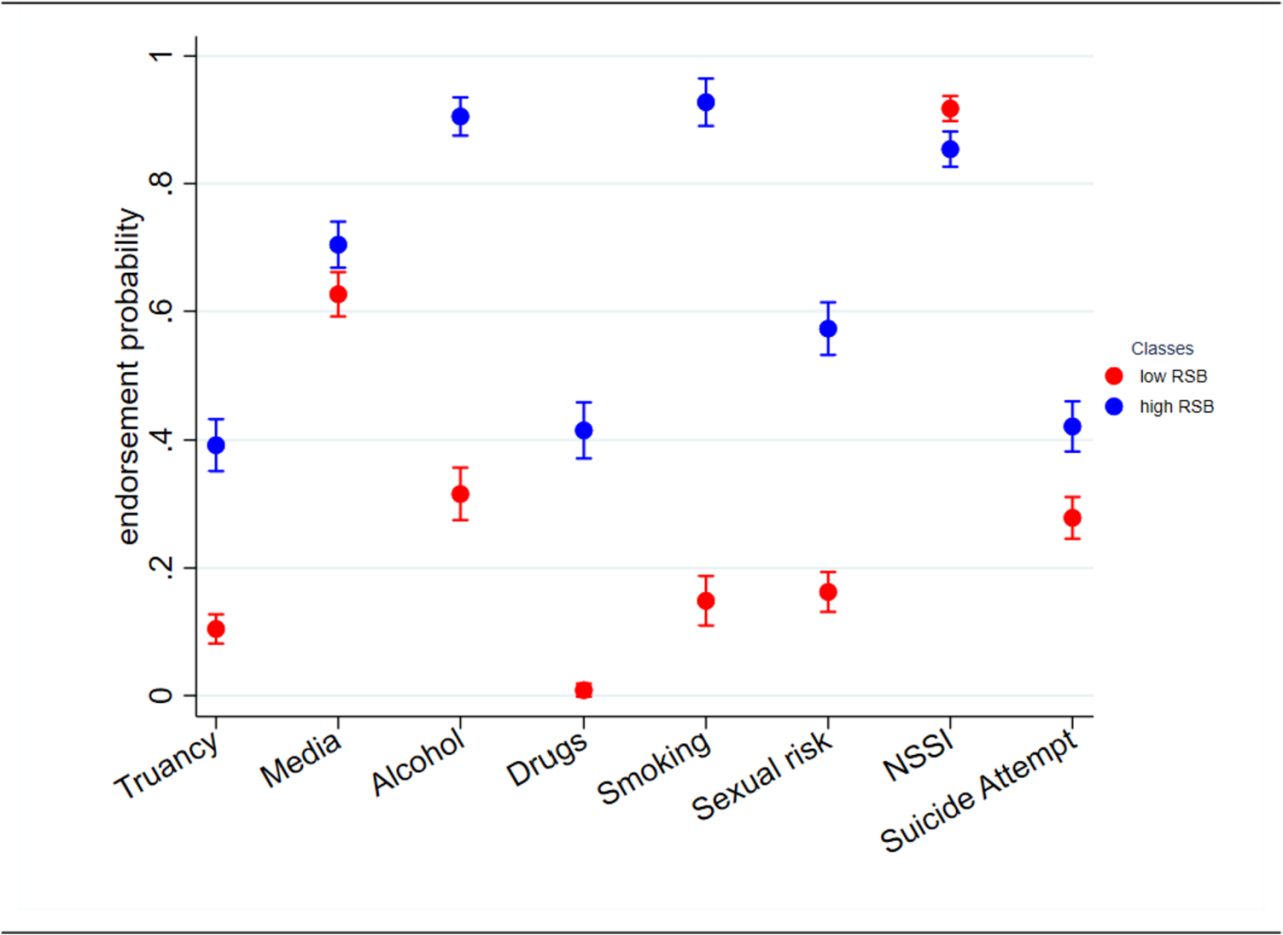
Two-class model of LCA with the probabilities for each RSB by class. Classes were labeled as “low RSB” and “high RSB” based on the general pattern of endorsement probabilities

Class 1 (hereinafter called *low RSB class*) comprised 54.7% (n = 222) of the sample and was characterized by adolescents with low endorsement probabilities for all RSB (estimated mean endorsement probabilities between *M* = 0.01 to *M* = 0.32), except from excessive media use (*M* = 0.63) and NSSI (*M* = 0.92). Class 2 (hereinafter called *high RSB class*) comprised 45.3% (n = 183) of the total sample and was characterized by adolescents with high endorsement probabilities for all RSB (endorsement probabilities between *M* = 0.39 to *M* = 0.93). Classes significantly differed in their probabilities for all RSB, except from excessive media use (χ^2^_(1)_ = 2.15, p = 0.708), NSSI (χ^2^_(1)_ = 3.23, *p* = 0.451), and suicide attempts (χ^2^_(1)_ = 6.98, *p* = 0.064; see Fig. 1 and Online Resource, Table E).

Belonging to the high RSB class was associated with a 2.62-fold increased likelihood of having a BPD diagnosis and increased the number of BPD criteria by 0.57 compared with belonging to the low RSB class. In contrast, the two classes did not differ in the likelihood of a diagnosis or in severity of depression (Table 4). When the analyses were repeated with age and sex as covariates, the results remained unchanged (see Online Resource, Table F).

**Table 4:** Univariate associations of BPD and depression diagnosis and severity with LCA classes

	Low RSB class		High RSB class		Class differences				
	M (SE)	95% CI	M (SE)	95% CI	OR	*β*	SE	95% CI	*p*^a^
BPD diagnosis	0.13 (0.03)	[0.08; 0.18]	0.26 (0.04)	[0.19; 0.34]	2.62		0.62	[1.65; 4.15]	< *0.001*
Number of BPD criteria	2.17 (0.12)	[1.96; 2.38]	3.16 (0.12)	[2.92; 3.40]		0.57	0.07	[0.43; 0.71]	< *0.001*
Diagnosis of depression	0.48 (0.43)	[0.40; 0.57]	0.42 (0.04)	[0.34; 0.50]	0.86		0.17	[0.58; 1.27]	*0.436*
Severity of depression	25.62 (0.81)	[24.03; 27.21]	25.94 (0.84)	[24.28; 27.60]		− 0.57	1.04	[− 2.61; 1.47]	*0.583*

All models without age and sex as covariates

*LCA* Latent Class Analysis; *OR*  odds ratio for high RSB class, *β* regression coefficient

^a^p-values were not adjusted for multiple testing

## Discussion

This study examined the associations of RSB with BPD and depression (diagnosis and severity) for the first time in a clinical sample of adolescents, extending the existing literature based primarily on non-clinical samples. Results show that higher frequencies of all RSB (except from excessive media use) were associated with a higher likelihood of BPD diagnosis and higher BPD severity. In contrast, only NSSI and suicide attempts were associated with a higher likelihood of a depression diagnosis and higher depression severity, whereas illicit drug use was associated with lower depression severity. The results remained largely unchanged when the analyses were adjusted for sex and age as covariates, the co-occurrence of BPD and depression, and the conceptual overlap between RSB and BPD. LCA resulted in a two-class solution, with classes differing in the likelihood of occurrence of all RSB except excessive media use, NSSI, and suicide attempts. The high RSB class showed a higher likelihood of a BPD diagnosis and of endorsing more BPD criteria, whereas no associations were found between the classes and depression diagnosis or severity.

In the current study, RSB were able to differentiate between typically highly comorbid BPD and depression, even in a clinical sample in which RSB are generally more common [28]. For instance, substance use (i.e., alcohol, drug, and tobacco use) was positively correlated with BPD pathology, while illicit drug use was negatively correlated with depression severity. These findings are consistent with the results of previous studies that BPD is associated with problematic alcohol use and illicit drug use, independently of anxiety and depressive symptomatology [19, 22, 38]. Thus, substance use may represent a maladaptive coping strategy to avoid negative emotions or to cope with stress associated with BPD symptoms [51, 52]. In contrast to adolescents with BPD pathology, depressed adolescents tend to withdraw themselves from social contacts and therefore lack the opportunity to experiment with substances in the peer context [53].

The finding suggesting sexual risk behavior to serve as specific marker for BPD pathology in adolescents is in line with previous evidence that BPD symptoms are linked to a higher number of romantic partners and that higher numbers of BPD symptoms at age 14 were associated with greater increases in sexual risk behavior over time, but not vice versa [23, 54]. Sexual risk behavior could represent an attempt to avoid partner rejection and abandonment or it may result from relationship instability associated with multiple sexual partners [27]. The underlying mechanisms are not yet fully understood. In addition, individuals with BPD were previously found to have higher rates of sexually transmitted infections compared with individuals without or with other personality disorders [23], suggesting that sexual risk behavior is not only an important early marker for preventing chronicity of BPD but also for preventing long-term sexual health risks. It should be noted, however, that the association between sexual risk behavior and BPD diagnosis disappeared when the models were adjusted for covariates.

Truancy showed similar effect sizes as sexual risk behavior. Likewise, a previous study found that individuals with BPD had more truancy days compared to individuals without or with other personality disorders [23]. Truancy could be an important early sign of BPD particularly as it is an easily identifiable behavior for both schoolteachers and educators. Therefore, a strong network between educational institutions such as schools and mental health services becomes even more important.

Excessive media use was the most common RSB in the entire sample, with a prevalence of 66%, but was not associated with BPD diagnosis and only weakly associated with BPD severity. In fact, there is limited evidence to date of the association between media use and BPD. A study on the longitudinal association between RSB and BPD found a significant association between onset of excessive media use (OR = 1.52) and subsequent BPD diagnosis, but this disappeared completely when models were adjusted for covariates [19]. In addition, previous findings showed an association between pathological Internet use (PIU) and several psychiatric disorders, including BPD [55]. The corresponding authors hypothesized that in patients with PIU, difficulties with identity might be compensated for by excessive Internet use, which could also apply to individuals with BPD. However, further studies are needed to assess the additional value of excessive media use as a risk marker for BPD and psychopathology in general.

In contrast to the other RSB studied, NSSI and suicide attempts demonstrated associations with both BPD and depression pathology. On one hand, this finding is reasonable, given that suicidal behavior is part of the diagnostic criteria according to DSM-5 for both BPD and depression. Additionally, NSSI is recognized as a strong predictor of future suicide attempts [56, 57] and serves as a critical transdiagnostic marker of psychological distress [58]. Although NSSI presents as one of the most common symptoms of BPD in adolescents [59], it has also be found to be a correlate of affective disorders [60] as well as a predictor of depression in particular, even though this relationship is not consistently observed [61–63]. On the other hand, the association of NSSI and suicide attempts with both, BPD and depression, raises the question of whether it is the result of the high comorbidity between BPD and depression. Our sensitivity analyses revealed that while NSSI demonstrated connections with both disorders, it was not linked to the co-occurrence of BPD and depression. In contrast, suicide attempts exhibited a heightened likelihood of the co-occurrence of BPD and depression; however, this probability was outweighed by the higher likelihood of experiencing depression alone. These findings underscore that the associations between NSSI or suicide attempts with BPD or depression are not solely attributable to the high comorbidity between the two disorders. Moreover, the other RSB studied showed no relationship with the co-occurrence of BPD and depression but displayed clear associations with BPD. This highlights the distinct significance of RSB for BPD, which is not explained by co-occurring depression.

The additional sensitivity analyses with age and sex as covariates led to three changes in the results: There was no longer an association between (1) sexual risk behavior and BPD diagnosis, (2) NSSI and depression diagnosis, and (3) illicit drug use and depression severity. In all cases, female sex and, in cases (1) and (2), older age were associated with the BPD and depression outcome variables, respectively. To some extent, the results certainly reflect the higher prevalence of BPD and depression in women in clinical samples, and thus the strong influence of sex in these two disorders [1], as well as the time it takes for a disorder to develop.

The fact that the high RSB class was associated with a higher likelihood of BPD diagnosis and more BPD criteria, while at the same time no associations were found between either class and depression pathology, suggests that the occurrence of risk behaviors, notably multiple risk behaviors, may be a specific indicator of BPD pathology. It should be noted that both classes had an average of BPD criteria that was below the diagnostic cut-off of 5 BPD criteria. However, previous studies have shown that subthreshold BPD (i.e., 3–4 BPD criteria) is already associated with significant impairment in quality of life and psychopathological distress compared with individuals without BPD (i.e., < 3 BPD criteria), which is not different from individuals with a full diagnosis [59]. Thus, the presence of various RSB may help to identify individuals who are at a very early, subclinical stage of BPD development and, therefore, being in need of indicated prevention [64, 65]. In addition, both groups showed a high likelihood of NSSI and suicide attempts, suggesting that these behaviors are more transdiagnostic in nature [60, 66, 67]. It appears that the occurrence of self-harm behaviors along with risk behaviors may be an indicator of evolving BPD pathology in adolescents.

### Clinical implication

Recording RSB may yield a valuable indication of the presence of BPD pathology in adolescents, especially when risk behaviors and self-harm behaviors co-occur. As some RSB, such as truancy, may be particularly well identified by teachers, schools and mental health services should work closely in the assessment of RSB to inform early intervention efforts.

### Limitations

First, due to the cross-sectional design of the study, no causal interpretation of the temporal associations between RSB and BPD or depression are warranted. Future studies should examine the impact of RSB on the developmental course of BPD and depression over time using longitudinal designs. Second, participants were predominantly female and collected from a specialized outpatient clinic for RSB, resulting in high prevalence rates of RSB in the sample. This limits the generalizability of the present findings to male participants and other psychiatric samples. Third, RSB that are clustered among male adolescents such as delinquent behavior [68] or risky driving [69] have not been assessed. Further studies are needed to examine RSB that may be early markers of BPD in male adolescents. Fourth, the thresholds for RSB are not consistent across the literature and were expert-based rather than evidence-based in the current study. To create consistent evidence, it would be necessary to examine the frequency and time frame in which a behavior must occur to be considered as RSB.

## Conclusion

Overall, the results suggest that the presence of self-harm behavior alone is associated with both BPD and depression and may, therefore, represent a somewhat transdiagnostic marker for psychopathology in general [60, 66, 67], whereas the co-occurrence of both self-harm and risk behaviors may be a specific marker for BPD pathology in adolescents. The findings are of particular relevance to clinical practice, as some of these behaviors are readily identifiable, increasing the chance of detecting BPD in adolescents at an early stage to prevent serious long-term consequences and chronicity.

### Supplementary Information

Below is the link to the electronic supplementary material.

Supplementary file1 (DOCX 80KB)

## Data Availability

Data a is available upon request from the corresponding author.
