# Supplementary material for: Risk-taking and self-harm behaviors as markers of adolescent borderline personality disorder
Source: Eur Child Adolesc Psychiatry. 2024 Jan 9;33(8):2743–53. doi: 10.1007/s00787-023-02353-y (PMC11272750; doi:10.1007/s00787-023-02353-y)
Supplement: Supplementary file 1 — Supplementary file1 (DOCX 80KB) [file 787_2023_2353_MOESM1_ESM.docx]

**Supplement to:**

**Risk-Taking and Self-Harm Behaviors as Markers of Adolescent Borderline Personality Disorder**

**European Child and Adolescent Psychiatry**

Yasmine Blaha^1^, Marialuisa Cavelti^1^, Stefan Lerch^1^, Annekatrin Steinhoff^1^, Julian Koenig^1,2^, Michael Kaess^1,3,*^

**Affiliation:**

^1^ University Hospital of Child and Adolescent Psychiatry and Psychotherapy, University of Bern, Bern, Switzerland

^2^ Department of Child and Adolescent Psychiatry, Psychosomatics and Psychotherapy, Faculty of Medicine and University Hospital Cologne, University of Cologne, Cologne, Germany

^3^ Department of Child and Adolescent Psychiatry, Centre for Psychosocial Medicine, University Hospital Heidelberg, Heidelberg, Germany

**^*^Corresponding Author Address:** Prof. Dr. med. Michael Kaess, M.D., University Hospital of Child and Adolescent Psychiatry and Psychotherapy, University of Bern, Bern, Stöckli, Bolligenstrasse 141c, 3000 Bern 60, Switzerland; E-Mail: [michael.kaess@upd.ch](mailto:michael.kaess@upd.ch)

| **Table A**  *Operationalization of RSB* | | | |  |
| --- | --- | --- | --- | --- |
| **Behavior** | **Risk Cut-Off** | **Question** | **Response** ^a^ | |
| Truancy | … being absent from school for 20% (≥1 month) or more during the past 6 months with less of these days absent being excused | *In the past 6 months, how many days have you been absent from school (excluding school holidays) / at work/training/internship/FSJ (excluding vacations)?* | ≤ 5%  10%  **20%**  **30%**  **40%**  **50%**  **60%**  **70%**  **80%**  **90%**  **100%** | |
|  | or | *How many days were excused?* | **none**  **25%**  **50%**  **75%**  all | |
|  | … being absent from school for at least 6-10 additional single hours during the past 6 months with less of these hours being excused. | *In the past 6 months, did you have any extra missing hours from School/Work/Training/Internship/FSJ?* | 0  1-5  **6-10**  **11-15**  **16-20**  **21-25**  **> 25** | |
|  |  | *How many of these missing hours were excused?* | **none**  **25%**  **50%**  **75%**  all | |
| Excessive media use | … being online for at least 5-6 hours during weekdays  or | *In the past year, how many hours on average were you engaged in computer games or other online activities on a weekday (Monday to Friday)?* | < 1 hour  1-2 hours  3-4 hours  **5-6** **hours**  **7-8 hours**  **> 8 hours** | |
|  | … being online for at least 5-6 hours during weekend/holidays | *In the past year, how many hours on average were you engaged in computer games or other online activities on a single day during the weekend / holidays / public holidays?* | < 1 hour  1-2 hours  3-4 hours  **5-6 hours**  **7-12 hours**  **> 12 hours** | |
| Alcohol use | … consuming alcohol on at least one day per month during the past year  or | *In the past year, on how many days did you drink at least one glass of alcohol?* | never  occasionally  **at least 1 once per month**  **at least once per week**  **2-3 times per week**  **almost daily**  **daily** | |
|  | … consuming alcohol at least 1-2 times during the past month  or | *In the past 30 days, on how many days did you drink at least one glass of alcohol?* | 0 days  **1-2 days**  **3-5 days**  **6-9 days**  **10-19 days**  **20-29 days**  **daily** | |
|  | … at least 3 events with consuming 3 glasses of alcohol within 3 hours during the past year | *In the past year, did you drink 3 or more alcoholic drinks in one day? And did you drink 3 or more alcoholic drinks within 3 hours at these times? And have you done this 3 times or more in the past year?* | no  **yes** | |
| Illicit drug use | … consuming illicit drugs at least occasionally during the past year  or | *In the past year, on how many days did you consume illicit drugs?* | never  **occasionally**  **at least 1 once per month**  **at least once per week**  **2-3 times per week**  **almost daily**  **daily** | |
|  | … consuming illicit drugs on at least 1-2 days during the past month | *In the past 30 days, on how many days did you consume [specified illicit substance] at least once?* | 0 days  **1-2 days**  **3-5 days**  **6-9 days**  **10-19 days**  **20-29 days**  **daily** | |
| Tobacco use | … using tobacco on at least one day per month during the past year  or | *In the past year, on how many days did you smoke tobacco?* | never  occasionally  **at least 1 once per month**  **at least once per week**  **2-3 times per week**  **almost daily**  **daily** | |
|  | … using tobacco on at least 1-2 days during the past month | *In the past 30 days, on how many days did you smoke tobacco at least once?* | 0 days  **1-2 days**  **3-5 days**  **6-9 days**  **10-19 days**  **20-29 days**  **daily** | |
| Sexual risk behavior | … having sex with at least 1 person during the past year  or | *In the past year, with how many different people have you had sexual intercourse?* | 0  **1**  **2-3**  **4-7**  **≥ 8** | |
|  | … having unprotected sex at least 1-2 times during the past year  or | *In the past year, how often have you had sex without using contraception?* | 0 times  **1-2 times**  **3-5 times**  **6-10 times**  **11-15 times**  **16-20 times**  **> 20 times** | |
|  | … having sex without condom at least 1-2 times during the past year | *In the past year, how often have you had sex without using a condom?* | 0 times  **1-2 times**  **3-5 times**  **6-10 times**  **11-15 times**  **16-20 times**  **> 20 time** | |
| NSSI | … at least 1 occasion of intentional non-suicidal self-injury during the past year | *In the past year, how many times have you engaged in NSSI?* | **Reported at least once** | |
| Suicide attempts | … having had at least one suicide attempt during the past 12 months | *In the past year, how many suicide attempts have you made?* | **Reported at least once** | |
| *Note.* ^a^The response categories in bold represent the cut-offs for the corresponding risk behaviors. | | | |  |

| **Table B**  *Separate univariate associations of RSB with BPD and depression diagnosis and severity, adjusted for covariates* | | | | | | | | | |
| --- | --- | --- | --- | --- | --- | --- | --- | --- | --- |
|  | Truancy | Excessive media use | Alcohol use | Illicit drug use | Smoking | Sexual risk behavior | NSSI | Suicide attempt |  |
| **BPD Diagnosis** |  |  |  |  |  |  |  |  |  |
| OR | 1.88 | 1.14 | 2.08 | 2.52 | 2.46 | 1.48 | 5.01 | 2.42 |  |
| 95% CI | [1.13; 3.15] | [.70;  1.87] | [1.23; 3.55] | [1.41; 4.49] | [1.52; 3.99] | [.92; 2.39] | [1.45; 17.25] | [1.51; 3.86] |  |
| *p*^a^ | ***.029*** | *.631* | ***.015*** | ***.005*** | ***.001*** | *.149*^b^ | ***.023*** | ***.001*** |  |
|  |  |  |  |  |  |  |  |  |  |
| **Number of BPD criteria** |  |  |  |  |  |  |  |  |  |
| OR | 1.49 | 1.20 | 1.55 | 1.90 | 1.68 | 1.41 | 2.73 | 1.84 |  |
| 95% CI | [1.26; 1.74] | [1.04; 1.40] | [1.33; 1.81] | [1.58; 2.27] | [1.46; 1.94] | [1.21; 1.63] | [2.05; 3.65] | [1.59; 2.12] |  |
| *p* | ***<.001*** | ***.028*** | ***<.001*** | ***<.001*** | ***<.001*** | ***<.001*** | ***<.001*** | ***<.001*** |  |
|  |  |  |  |  |  |  |  |  |  |
| **Diagnosis of depression** |  |  |  |  |  |  |  |  |  |
| OR | 1.10 | 1.07 | .87 | .75 | .68 | .66 | 2.18 | 1.88 |  |
| 95% CI | [.68; 1.76] | [.70;  1.63] | [.56; 1.35] | [.44; 1.29] | [.45; 1.02] | [.42; 1.02] | [1.06; 4.47] | [1.23; 2.88] |  |
| *p* | *.726* | *.756* | *.581* | *.371* | *.096* | *.101* | *.061*^c^ | ***.009*** |  |
|  |  |  |  |  |  |  |  |  |  |
| **Severity of depression** |  |  |  |  |  |  |  |  |  |
| *β* | 1.64 | 1.68 | .89 | -2.55 | .95 | -1.37 | 7.48 | 3.22 |  |
| 95% CI | [-.69; 3.98] | [-.31; 3.68] | [-1.20; 2.99] | [-5.23; .14] | [-1.00; 2.90] | [-3.48; .74] | [4.20; 1.76] | [1.20; 5.23] |  |
| *p* | *.222* | *.143* | *.459* | *.100*^d^ | *.402* | *.258* | ***<.001*** | ***.005*** |  |
| *Note.* All models were run with age and sex as covariates; ^a^Benjamini-Hochberg corrected p-values for multiple testing; Adjustment for covariates yielded the following changes in the results: ^b^Sexual risk behavior was no longer associated with a BPD diagnosis (*p* = .149; OR_(age)_ = 1.32, *p_(age)_ = .002*; OR_sex(female)_ = 2.56, *p*_sex(female)_ *= .009*); ^c^NSSI was no longer associated with the diagnosis of depression (*p* = .061; OR_(age)_ = 1.18, *p*_(age)_ *= .022*; OR_sex(female)_= 1.87, *p*_sex(female)_ =*.036*); ^d^Illicit drug use was no longer associated with depression severity *(p* = .100; OR_sex(female)_ = 8.20, *p*_sex(female)_ *<.001*). | | | | | | | | | |

| **Table C**  *Effect of RSB on the comorbid occurrence of BPD and depression diagnoses based on bivariate logistic regression* | | | | | | | | |
| --- | --- | --- | --- | --- | --- | --- | --- | --- |
|  | **Truancy** | **Excessive media use** | **Alcohol use** | **Illicit drug use** | **Smoking** | **Sexual risk behavior** | **NSSI** | **Suicide attempt** |
| $\frac{COR(RSB=present)}{COR(RSB=absent)}$ | .83 | 1.26 | .53 | 1.01 | .73 | 2.25 | .23 | .27 |
|  |  |  |  |  |  |  |  |  |
| 95% CI | [.30; 2.24] | [.48; 3.30] | [.19; 1.48] | [.35; 2.90] | [.28; 1.93] | [.89; 5.68] | [.02; 2.94] | [.10; .69] |
| *p^a^* | *.807* | *.855* | *.605* | *.984* | *.842* | *.348* | *.520* | ***.048*** |
| *Note. ^a^*Benjamini-Hochberg corrected *p*-values for multiple testing. All models without age and sex as covariates. | | | | | | | | |

| **Figure A**  *Probabilities for combinations of BPD and depression diagnoses and suicide attempts.* |
| --- |
| 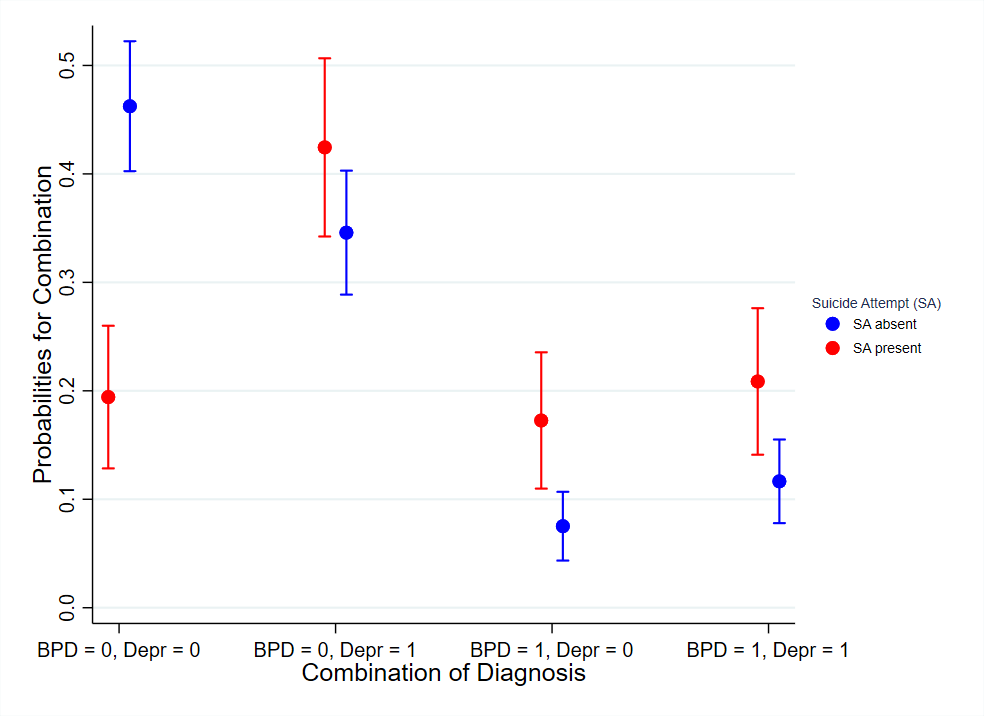 |
| *Note.* SA = Suicide Attempt. BPD / Depr = 0: Respective diagnosis is absent. BPD / Depr = 1: Respective diagnosis is present. Probabilities describe the likelihood of the occurrence of specific combination of diagnoses, given the condition that at least one suicide attempt was present / absent during the past year. |

| **Table D**  *Separate univariate associations of RSB with BPD diagnosis and severity with adjusted BPD severity for conceptual overlap between diagnostic BPD criteria and RSB* | | | | | | | | | |  |
| --- | --- | --- | --- | --- | --- | --- | --- | --- | --- | --- |
|  | **Truancy** | **Excessive media use** | **Alcohol use** | **Illicit drug use** | **Smoking** | **Sexual risk behavior** | | **NSSI** | **Suicide attempt** | |
|  |  |  |  |  |  |  |  | |  | |
| **Number of BPD criteria** (no impulsivity) |  |  |  |  |  |  |  | |  | |
| *β* |  |  | .47 | .37 |  | .32 |  | |  | |
| 95% CI |  |  | [.32; .62] | [.19; .54] |  | [.16; .47] |  | |  | |
| *p^b^* |  |  | ***<.001*** | ***<.001*** |  | ***<.001*** |  | |  | |
|  |  |  |  |  |  |  |  | |  | |
| **Number of BPD criteria** (no self-harm) |  |  |  |  |  |  |  | |  | |
| *β* |  |  |  |  |  |  | .76 | | .63 | |
| 95% CI |  |  |  |  |  |  | [.48; 1.04] | | [.47; .79] | |
| *p^b^* |  |  |  |  |  |  | ***<.001*** | | ***<.001*** | |
|  |  |  |  |  |  |  |  | |  | |
| *Note.* ^a^Benjamini-Hochberg corrected p-values for multiple testing; ^b^p-value has not been adjusted for multiple testing. All models without adjustment for age and sex. | | | | | | | | | |  |

| **Table E**  *Two-class model of LCA with the probabilities for each RSB by class* | | | | | | | |
| --- | --- | --- | --- | --- | --- | --- | --- |
|  | Low RSB class | | | High RSB class | | | *Group differences* |
|  | Mean | SE | 95% CI | Mean | SE | 95% CI | *p*^a^ |
| School | .10 | .02 | [.07; .16] | .39 | .04 | [.32; .47] | ***<.001*** |
| Media | .63 | .03 | [.56; .69] | .70 | .04 | [.63; .77] | *.706* |
| Alcohol | .32 | .04 | [.24; .40] | .90 | .03 | [.83; .95] | ***<.001*** |
| Illicit drugs | .01 | .01 | [.00; .08] | .42 | .04 | [.33; .50] | ***<.001*** |
| Smoking | .15 | .04 | [.09; .24] | .93 | .04 | [.81; .97] | ***<.001*** |
| Sex risk | .16 | .03 | [.11; .23] | .58 | .04 | [.49; .65] | ***<.001*** |
| NSSI | .92 | .02 | [.87; .95] | .85 | .03 | [.79; .90] | *.451* |
| Suicide Attempts | .28 | .03 | [.22; .35] | .12 | .04 | [.35; .50] | *.064* |
| *Note.* All models without age and sex as covariates; LCA = Latent Class Analysis; RSB = Risk-taking and self-harm behavior; ^a^Šidák-corrected p-values for multiple testing. | | | | | | | |

| **Table F**  *Univariate associations of BPD and depression diagnosis and severity with LCA classes adjusted for covariates* | | | | | | | | | | | | |
| --- | --- | --- | --- | --- | --- | --- | --- | --- | --- | --- | --- | --- |
|  | Low RSB class | | High RSB class | | | Models with age and sex as covariates | | | | | |  |
|  | M (SE) | CI | M (SE) | CI | OR | | *β* | SE | CI | *p*^a^ |  |  |
| BPD diagnosis | .13 (.03) | [.08; .18] | .26 (.04) | [.19; .34] | 2.45 | |  | .61 | [1.51; 3.99] | ***<.001*** |  |  |
| Number of BPD criteria | 2.17 (.12) | [1.96; 2.38] | 3.16 (.12) | [2.92; 3.40] |  | | .53 | .07 | [.39; .68] | ***<.001*** |  |  |
| Diagnosis of depression | .48 (.43) | [.40; .57] | .42 (.04) | [.34; .50] | .78 | |  | .17 | [.51; 1.19] | *.248* |  |  |
| Severity of depression | 25.62 (.81) | [24.03; 27.21] | 25.94 (.84) | [24.28; 27.60] |  | | .32 | 1.03 | [-1.71; 2.35] | *.756* |  |  |
| *Note.* All models without age and sex as covariate; LCA = Latent Class Analysis; OR = odds ratio for high RSB class; *β* = regression coefficient; ^a^p-values were not adjusted for multiple testing. | | | | | | | | | | | | |
